# Thrombotic microangiopathy with severe renal failure in adenocarcinoma

**DOI:** 10.4103/0971-4065.42342

**Published:** 2008-04

**Authors:** G. Lakshminarayana, R. Rajesh, N. V. Seethalekshmy, G. Kurian, V. N. Unni

**Affiliations:** Department of Nephrology, Amrita Institute of Medical Sciences and Research, Kochi, India; 1 Department of Pathology, Amrita Institute of Medical Sciences and Research, Kochi, India

**Keywords:** Gastric adenocarcinoma, thrombotic microangiopathy

## Abstract

Kidney disease frequently complicates malignancy and its treatment. The spectrum of renal disease in cancers includes acute kidney injury, chronic kidney disease and tubular disorders. Thrombotic microangiopathy (TMA) is an uncommon initial clinical presentation of malignancies. Renal failure is an extremely rare feature of cancer-associated TMA syndromes in the absence of chemotherapy. Here, we report a patient who presented to the hospital for the first time with TMA and severe renal failure requiring hemodialysis and was diagnosed with gastric adenocarcinoma.

Kidney disease frequently complicates malignancy and its treatment. Thrombotic microangiopathy (TMA), encountered in only approximately 5.7% of patients with advanced cancers, is an uncommon cause of acute renal failure (ARF).^1^ The clinical presentation of TMA is abrupt and often severe hemolytic anemia characterized by fragmented erythrocytes and thrombocytopenia in the peripheral blood and varying signs of organ injury due to the formation of thrombi in microcirculation. TMA has been reported in mucinous adenocarcinoma, the most frequent sites being the stomach, lung and breast.^1^,^2^ Unless it is identified and treated early, TMA is life-threatening and has a poor prognosis.

## Case Report

A 69-year-old man presented with severe weakness and fatigue for two week duration. He had oliguria, anorexia and nausea; he was not diabetic or hypertensive. There was no history of fever, diarrhea or any drug intake. On examination, he was fully conscious and oriented; had severe pallor, mild icterus, few ecchymotic patches on the skin and bilateral pedal edema; blood pressure was 160/106 mmHg; systemic examination including optic fundi did not reveal any abnormality.

**Fig. 1:**
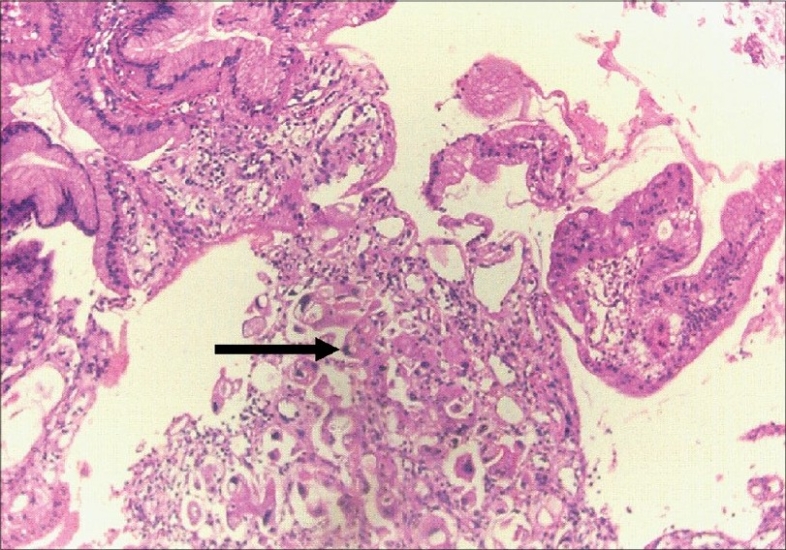
Gastric biopsy showing moderately differentiated adenocarcinom (H&E, ×150)

**Fig. 2:**
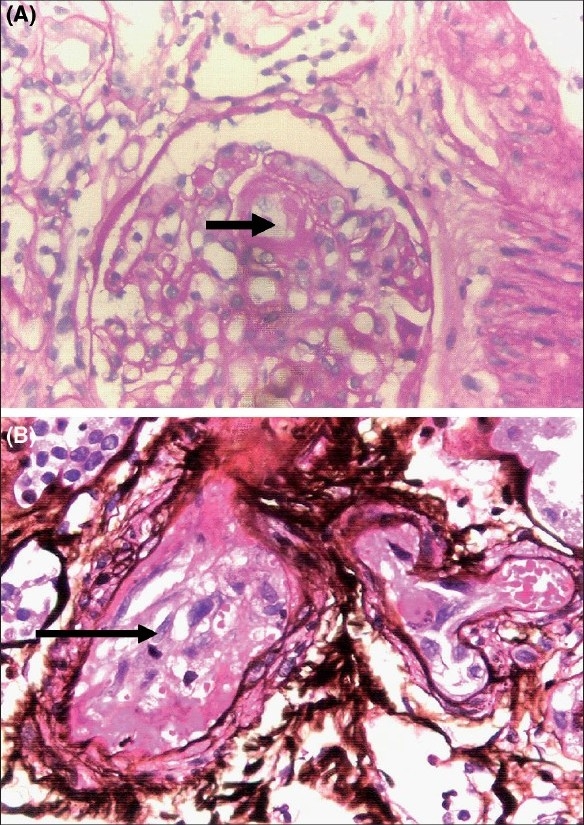
(A) Kidney biopsy showing fibrin thrombi (arrow) in the capillary lumen (periodic acid-Schiff (PAS) stain, ×400). (B) Kidney biopsy showing fibrin thrombi (arrow) in a medium sized vessel. (silver methenamine stain, ×400)

Investigations showed hemoglobin of 6.5 g/dl with normal white blood corpuscle (WBC) counts (total and differential), mean corpuscular volume (MCV) and mean corpuscular hemoglobin (MCH); platelet count, 64,000/mm^3^; peripheral blood smear showed thrombocytopenia and normocytic-normochromic anemia with fragmented erythrocytes, schistocytes, helmet cells and polychromatophilia, suggestive of hemolysis. Corrected retic count was 4.5%. Coombs test (direct and indirect) was negative. Total bilirubin was 3.4 mg/dl (indirect, 2.4 mg/dl); serum LDH, 914 IU/l; serum proteins and transaminases, fibrinogen, prothrombin time and activated partial thromboplastin time (APTT), normal. Serum creatinine was 5.9 mg/dl and blood urea 150 mg/dl with normal serum electrolytes. Urine showed 2 + proteinuria and microscopy revealed 6 - 8 red blood corpuscles (RBCs)/high-power field (HPF); 24-h urine protein was 1.4 g. Blood and urine cultures did not reveal any growth, and serum complements were normal. The renal functions deteriorated after admission, and hemodialysis was initiated. An upper gastrointestinal (GI) endoscopy was performed, and it revealed a giant gastric ulcer; biopsy [Fig. 1] showed moderately differentiated adenocarcinoma. Kidney biopsy was performed after hemodialysis and blood transfusions. Light microscopy [Fig. 2] showed glomeruli with fibrin thrombi in the capillary lumen. Tubules showed mild regenerating changes with the hyaline cast and RBCs in the lumen. Interlobular arteries showed intimal thickening with the narrowing of the lumen; fibrinoid necrosis and luminal thrombi were also noted; arterioles showed fibrinoid necrosis and luminal thrombi. Immunofluorscence showed glomeruli with fibrin positivity in the vessels; was negative for immunoglobulin (Ig)G, IgM, IgA, complement (C)3 and C1q. He was diagnosed as having gastric adenocarcinoma with TMA and ARF.

He was initiated on daily plasmapheresis (9 sessions administered with plasma removal of 40 ml/kg body weight at each session); hemodialysis was continued. Subsequently, he underwent subtotal gastrectomy, after which plasmapheresis was continued on alternate days for six more sessions. Hemolysis subsided; platelet count and serum LDH levels became normal in approximately two weeks after gastrectomy. Plasmapheresis was discontinued, and the patient was continued on hemodialysis on alternate days. He was discharged with an advice to continue on hemodialysis and observe any improvement in the renal function. He continued to be dependent on dialysis at the end of six months.

## Discussion

TMA describes a set of pathological changes, which include intrarenal or systemic microvascular thrombi with endothelial swelling and microvascular obstruction. There is no consensus on the diagnostic criteria for TMA syndrome; nevertheless, laboratory features include microangiopathic hemolytic anemia and thrombocytopenia although renal failure, neurological abnormalities and gastrointestinal symptoms are common.^1^

TMA syndromes are known to be a complication of the tumor state itself and of certain treatment regimens.^1^ TMA has most commonly been associated with carcinomas. First described by Brain *et al* in 1962, 75% of TMA occur in association with gastric, breast and lung cancers.

A^1^–^3^ prospective study determined that 5.7% of patients with metastatic carcinoma have microangiopathic hemolytic anemia.^1^ Gastric carcinoma accounts for more than half of the cases, followed by breast and lung carcinomas. Renal failure is an uncommon feature of cancer-associated TMA syndromes in the absence of chemotherapy.^1^ Table 1 summarizes the causes of TMA.

**Table 1:** Causes of thrombotic microangiopathies (TMA)

I.	Infections Viral infections: Coxsackie virus, echovirus, adenovirus, human immunodeficiency virus (HIV) Bacterial infections: *Shigella, Salmonella, Streptococcus* sp., *Escherichia coli*
II.	Systemic diseases Malignant hypertension Systemic lupus erythematosus and APLA syndrome Scleroderma Malignancies of stomach, breast and lung
III.	Pregnancy associated: Pregnant or postpartum states
IV.	Drugs: Mitomycin C, Bleomycin, Cisplatin, Gemcitabine, Deoxycoformycin, Methyl CCNU, Gemcitabine, Daunorubicin, Cytosine Arabinoside, Ticlopidine, Clopidogrel, Quinine, Calcineurin inhibitors, OKT3 oral contraceptives.
V.	Genetic or immune-mediated abnormalities of the complement system Factor H deficiency Membrane cofactor protein abnormalities

The pathogenesis of TMA is still not well understood. Recent studies, however, have shed some light on the understanding of the pathophysiological process. Relatively consistent findings have been the deficiency of a von Willebrand factor (vWF)-cleaving protease (A Disintegrin And Metalloprotease with a ThromboSpondin type 1 domain 13 (ADAMTS 13)) due to an inhibitor(s) or a congenital deficiency. This leads to the presence of unusually large vWF multimers in TMA patients. It is known that the protease cleaves these large vWF multimers into smaller ones. Unusually large vWF multimers, if not cleaved, promote the activation and aggregation of platelets. In a disease or injury involving the endothelial cell, unusually large vWF multimers may be released into the circulation; further, these multimers, in the absence of vWF-cleaving protease, promote the activation and aggregation of platelets in the arterioles and capillaries, resulting in thrombocytopenia and TMA.^5^

It is speculated that in advanced cancers, in addition to abnormal angiogenesis in the marrow, aggressive growth of tumors and secondary myelofibrosis may injure endothelial cells in the vessels in the marrow by direct invasion. These changes may cause the release of large vWF multimers and a possible decrease in the availability of the unusually large vWF-cleaving protease through undetermined mechanisms (decreased production or immune reaction), and may contribute to the aggregation of platelets.^5^ Since TMA has been mostly observed in adenocarcinoma, this pathology could be another contributing factor, possibly associated with the production of mucin, which may exert a direct detrimental effect on the pathological endothelial cell and alter the endothelial function.^5^

If untreated, TMA in adults typically follows a progressive course in which irreversible renal failure and death are common outcomes. The mortality rate prior to the use of plasma exchange was as high as 90%.

Plasma therapy (plasma exchange and plasma infusion) has been reported to show 60 - 80% response rates and patient survival rates over 90% in TMA. Plasma exchange is efficacious because not only is the missing factor replenished but also the toxic substances are removed during the exchange procedure.^4^ A recent prospective trial comparing the response in TMA patients to either plasma infusion or plasma exchange revealed a 78% successful response rate to plasma exchange and only a 31% response rate to plasma infusion alone.^2^ Patients who failed to respond to plasma infusion initially and were subsequently treated with plasma exchange also achieved a high rate of response (78%), suggesting that plasma exchange therapy is more effective than plasma infusion alone. Many different schedules have been reported for plasma exchange, but most authors recommend an initial 7 d of therapy followed by alternate-day treatment until hematological improvement is observed (platelet count, hemoglobin and serum lactate dehydrogenase (LDH) are useful parameters to monitor). Recommended exchange volumes are 1-1.5 times the predicted plasma volume.^2^

## Summary

Renal failure remains an important complication in cancer and its treatment. A simple and systematic approach to assess and treat potential prerenal, intrarenal and postrenal causes is indicated in all patients. Early diagnosis and treatment of renal failure is vital to the improvement of renal outcomes and to ensure that patients are best prepared for further treatment of the malignancy.

TMA represents a spectrum of diseases consisting of multiple etiologies that has a final common pathway of multiorgan microvascular thrombosis. If untreated, it has been associated with very high morbidity and mortality rates. Thus far, several modes of therapy have been used, with plasma exchange being the most effective method studied and shown to improve patient survival. Although many of the patients recover and attain normal renal function, those with severe TMA may develop sclerosis with residual hypertension and chronic renal impairment requiring continuation of renal replacement therapy.

Our patient with gastric adenocarcinoma presented to the hospital for the first time with TMA and severe renal failure requiring dialysis, which is an extremely rare presentation.
